# Circadian rhythms-related disorders in diurnal fat sand rats under modern lifestyle conditions: A review

**DOI:** 10.3389/fphys.2022.963449

**Published:** 2022-09-07

**Authors:** Carmel Bilu, Haim Einat, Paul Zimmet, Noga Kronfeld-Schor

**Affiliations:** ^1^ School of Zoology, Tel-Aviv University, Tel Aviv, Israel; ^2^ School of Behavioral Sciences, Tel Aviv-Yaffo Academic College, Tel-Aviv, Israel; ^3^ Department of Diabetes, Monash University, Melbourne, VIC, Australia

**Keywords:** circadian desynchrony, circadian rhythms, fat sand rats, diurnality, diabetes, depression

## Abstract

Modern lifestyle reduces environmental rhythmicity and may lead to circadian desynchrony. We are exposed to poor day-time lighting indoors and excessive night-time artificial light. We use air-conditioning to reduce ambient temperature cycle, and food is regularly available at all times. These disruptions of daily rhythms may lead to type 2 diabetes mellitus (T2DM), obesity, cardiometabolic diseases (CMD), depression and anxiety, all of which impose major public health and economic burden on societies. Therefore, we need appropriate animal models to gain a better understanding of their etiologic mechanisms, prevention, and management.We argue that the fat sand rat (*Psammomys obesus*), a diurnal animal model, is most suitable for studying the effects of modern-life conditions. Numerous attributes make it an excellent model to study human health disorders including T2DM, CMD, depression and anxiety. Here we review a comprehensive series of studies we and others conducted, utilizing the fat sand rat to study the underlying interactions between biological rhythms and health. Understanding these interactions will help deciphering the biological basis of these diseases, which often occur concurrently. We found that when kept in the laboratory (compared with natural and semi-wild outdoors conditions where they are diurnal), fat sand rats show low amplitude, nocturnal or arrhythmic activity patterns, dampened daily glucose rhythm, glucose intolerance, obesity and decreased survival rates. Short photoperiod acclimation exacerbates these pathologies and further dampens behavioral and molecular daily rhythms, resulting in CMD, T2DM, obesity, adipocyte dysfunction, cataracts, depression and anxiety. Increasing environmental rhythmicity by morning bright light exposure or by access to running wheels strengthens daily rhythms, and results in higher peak-to-trough difference in activity, better rhythmicity in clock genes expression, lower blood glucose and insulin levels, improved glucose tolerance, lower body and heart weight, and lower anxiety and depression. In summary, we have demonstrated that fat sand rats living under the correspondent of “human modern lifestyle” conditions exhibit dampened behavioral and biological rhythms and develop circadian desynchrony, which leads to what we have named “The Circadian Syndrome”. Environmental manipulations that increase rhythmicity result in improvement or prevention of these pathologies. Similar interventions in human subjects could have the same positive results and further research on this should be undertaken.

## Introduction

The circadian system is a major regulator of almost every aspect of the body (Kuhlman et al., 2018; Top and Young, 2018). The master ‘Body Clock’ resides within the suprachiasmatic nucleus (SCN) of the hypothalamus and determines daily rhythms in physiology and behavior through the synchronization of peripheral clocks in nearly all cells in the body, including the body’s key tissues such as the brain, heart, liver, muscle and adipose tissue (Dardente et al., 2007; Dibner et al., 2011; Kalsbeek et al., 2011; Honma, 2020; Jouffe et al., 2022).

Light is the primary environmental signal that entrains the main circadian clock in the SCN, discriminating day from night and synchronizing the transcription - translation feedback loop of genes involved in the internal clock function (Challet and Pevet, 2003). In turn, the SCN affects circadian physiology and behavior via humoral and neuronal cues and by synchronization of local oscillators in the cells of most organs and tissues (Dibner et al., 2011). Peripheral clocks are also directly and indirectly affected by other environmental factors such as temperature change and food intake (Li et al., 2012; Orozco-Solis and Sassone-Corsi, 2014; Zimmet et al., 2019).

Modern life reduces the environmental rhythmicity that humans are exposed to (Wyse et al., 2014; West and Bechtold, 2015). Electrical light and staying indoors decreases the amplitude of light exposure and effects other daily rhythms in human lifestyle by enabling activity to occur during hours of natural darkness (Wyse et al., 2014). Other environmental cycling cues are also affected by the wide-spread use of electricity, such as the use of air-conditioning and heating systems to reduce ambient temperature and humidity cycles (Kanikowska et al., 2015), and the increase in food availability is allowing calorie-rich diet consumption at any time (Engin, 2017). These modern life conditions strongly influence our biological rhythms and health.

One of the most fundamental features of a species is its activity pattern, which requires anatomical, physiological, and behavioral adaptations. Early mammals were small nocturnally active insectivores (Gerkema et al., 2013). All modern mammals developed from this nocturnal common ancestor, and most of them maintained a robust and relatively homogenous nocturnal activity pattern (Kronfeld-Schor and Dayan, 2008). However, throughout evolution, mammals from independent evolutionary lineages switched to a diurnal activity pattern (Roll et al., 2006). This shift, from the ancestral nocturnal activity pattern to the current diurnal one, required morphological, physiological, and behavioral adaptations including adaptations of the circadian system. This occurred independently among these species, in a convergent manner, making the diurnal circadian system less homologous and more diverse than the nocturnal one (Kronfeld-Schor and Dayan, 2008; Hut et al., 2012).

The precise mechanisms defining the circadian system as nocturnal or diurnal are still unknown. There are no fundamental differences distinguishing the SCN of diurnal species from those of nocturnal ones (Smale et al., 2003; Schwartz et al., 2004; Lambert et al., 2005; Cohen et al., 2010). For example, expression of genes that are central to the core molecular oscillator, production of SCN output signals such as vasopressin, as well as rhythms of metabolic activity in the SCN, are the same in diurnal and nocturnal species (Smale et al., 2008; Cohen et al., 2010). In addition, melatonin, the main hormonal signal of the circadian system, is secreted during the night, and it is secretion is inhibited by light in both diurnal and nocturnal species (Armstrong, 1989). Because of these similarities, it had been suggested that the differences in the circadian function of diurnal and nocturnal species are not found in the SCN itself but rather in the interpretation of the SCN signals (Smale et al., 2003; Lee, 2004; Hagenauer and Lee, 2008). This means that in order to switch from the ancestral nocturnal activity pattern to a diurnal one, the downstream interpretation of the signals emanating from the SCN and melatonin needs to be reversed (Smale et al., 2008; Cuesta et al., 2009; Kumar Jha et al., 2015). Furthermore, to become diurnal, the species should first eliminate all those functions that contributed to confining and synchronizing the activity of the nocturnal ancestor to the nocturnal phase and enhance the robustness of the nocturnal activity pattern (e.g., masking effects of light). In the second stage, the species should reverse these functions to develop a high robustness of diurnal rhythms (as seen in nocturnal mammals).

Indeed, melatonin has opposite physiological and behavioral effects in nocturnal and diurnal species. In diurnal species, the nocturnal secretion of melatonin is concurrent with the quiescence phase (sleepiness and decreased locomotor activity and body temperature). Yet in nocturnal species, it is related to vigilance and to an increase in body temperature and locomotor activity (Cagnacci et al., 1992; Huber et al., 1998; Zisapel et al., 1998; Arendt, 2000; Zhdanova and Giorgetti, 2002; Aparicio et al., 2006; Hagenauer and Lee, 2008; Bilu and Kronfeld-Schor, 2013). Further, melatonin treatment promotes sleep and reduces activity levels and body temperature in diurnal species whereas in nocturnal species, it increases alertness, locomotor activity, and body temperature (Mendelson et al., 1980; Bartness et al., 1993; Dollins et al., 1994; Roseboom et al., 1996; Huber et al., 1998; Zisapel et al., 1998; Arendt, 2000; Mendelson, 2002; Bilu and Kronfeld-Schor, 2013). Daily rhythms in the periphery are also usually reversed in diurnal compared to nocturnal species (Yan et al., 2020). For example, in the diurnal olive baboon (*Papio anubis*), the expression of core clock genes in the periphery is in-phase with the SCN, but around 12 h out of phase in other brain or peripheral tissues (Mure et al., 2018). This is consistent with findings from other diurnal species such as Nile grass rats and humans (Ramanathan et al., 2008a; Ramanathan et al., 2008b; Li et al., 2013; Yan et al., 2020).

It was previously shown that the reversal of the functions that contributed to confining and synchronizing the activity of the nocturnal ancestor to the nocturnal phase have occurred to a limited extent in various diurnal rodents, resulting in a reduction in the robustness of the circadian system of diurnal species (Cohen et al., 2010; Barak and Kronfeld-Schor, 2013; Bilu and Kronfeld-Schor, 2013; Bilu et al., 2016; Bilu et al., 2019a; Bilu et al., 2019b). In fact, when kept under laboratory conditions where low intensity light is the only cycling variable, many diurnal rodents tested to date demonstrate an unstable nocturnal phase preference, with low amplitude, and in some cases, no rhythm at all. Many of them show some form of rhythm instability, and their activity is not completely confined to the light phase (Fat sand rats (Barak and Kronfeld-Schor, 2013; Touati et al., 2018; Bilu et al., 2019a), golden spiny mice (Cohen and Kronfeld-Schor, 2006), Nile grass rats (Blanchong et al., 1999), degus (Hagenauer and Lee, 2008), tuco-tuco (Tomotani et al., 2012), Mongolian gerbil (Umezu et al., 1989)).

Such a response to laboratory conditions acclimation has not been documented in nocturnal rodents (Weber and Hohn, 2005; Barak and Kronfeld-Schor, 2013; Shankar and Williams, 2021). We hypothesize that this observed reduction in the robustness of the circadian system of diurnal species increases their susceptibility to circadian rhythm-related disturbances and disorders. We further suggest, as mentioned later, that this tendency leads diurnal species to develop the Metabolic Syndrome, and because of the circadian element, we have proposed renaming it the Circadian Syndrome (Zimmet et al., 2019).

Considering that the circadian systems of nocturnal and diurnal mammals differ significantly via an as yet unknown mechanism, it is surprising that most biomedical research regarding disorders in diurnal humans, including those related to the circadian system, has been performed using nocturnal animals such as laboratory mice and rats. In the literature about the development of animal models it was repeatedly noted that the selection of model organisms for most studies puts significant constrains on research and must be acknowledged and addressed (Bolker, 2012). Moreover, significant work in the field of animal modeling demonstrated the advantages of selecting models with high homology to the modeled human system. In that context, we have previously suggested that using diurnal model animals may very well be advantageous compared with nocturnal models in research that aims to understand the underlying mechanisms of the interactions between circadian rhythms and disease (Bilu et al., 2016). Here, we review our studies in the fat sand rat (*Psammomys obesus*) supporting the utilization of a diurnal model animal for circadian rhythm related diseases, including Type 2 diabetes mellitus, obesity, cardiometabolic and mental health conditions.

### The fat sand rat (*Psammomys obesus*)

**FIGURE 1:**
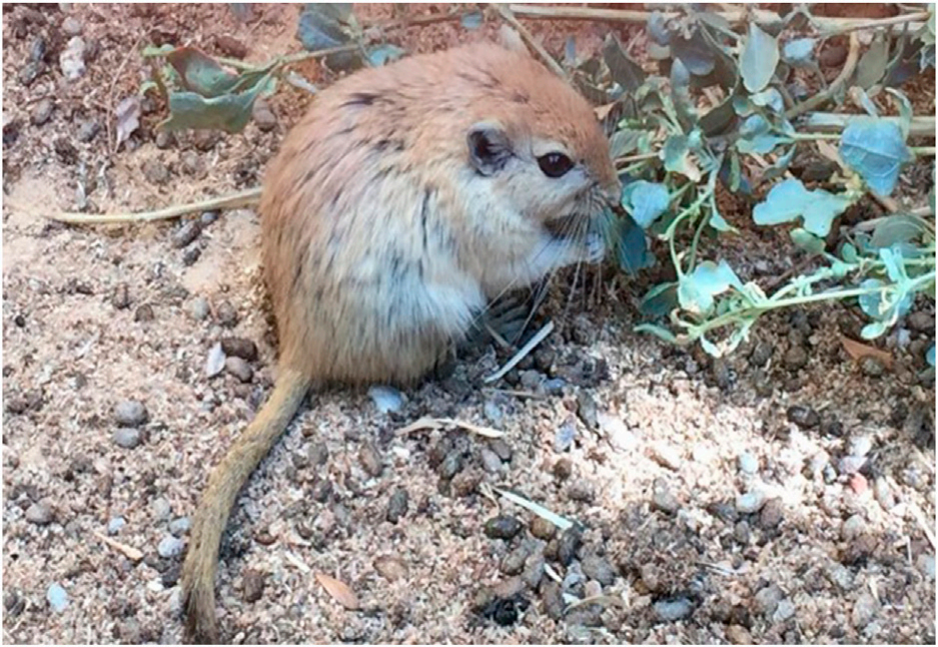
The fat sand rat (*Psammomys obesus*) feeding on its favorite diet, saltbush (*Atriplex halimus*).

The fat sand rat (see Figure 1) is a large burrow-dwelling gerbil (160 ± 30 g) that inhabits wadi beds, saline and saline-marsh plains in the deserts of North Africa, from Mauritania to Egypt, Sudan and Israel (Mendelssohn Yom-Tov, 1999). They feed mostly on saltbush (*Atriplex halimus*), a plant relatively low in energy content and high in ash and water (Degen et al., 1988).

In the wild, fat sand rats are strictly diurnal. The above-ground activity in nature includes both feeding and food caching (Tchabovsky et al., 2001a; Tchabovsky et al., 2001b; Gromov, 2001; Tchabovsky and Krasnov, 2002). In summer, they are active above ground mostly during early hours of the morning and in the afternoon, avoiding activity during the hot summer mid-day hours. During winter time, they are active for about 5 h during mid-day (Ilan Yom-Tov, 1990; Barak and Kronfeld-Schor, 2013).

**FIGURE 2:**
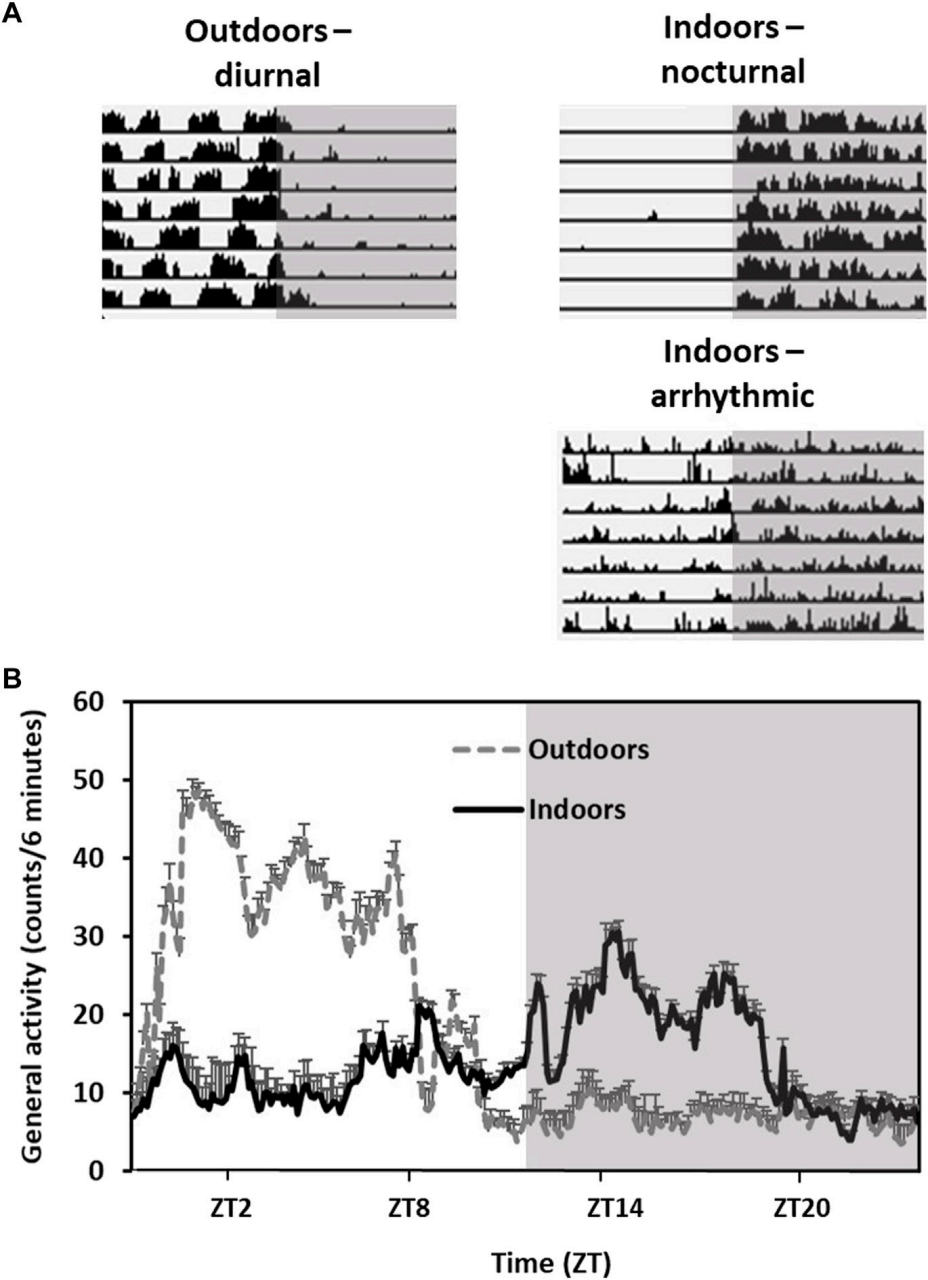
Activity rhythms of fat sand rats kept outdoors *vs.* indoors in the same laboratory cages. Grey background represents dark hours. **(A)** Representative daily actograms of an animal kept outdoors (diurnal) and two animals kept indoors (nocturnal and arhythmic). Each row represents 1 day. Days are depicted one below the other. **(B)** Activity rhythm: Data are the mean ± SEM of daily activity arrhythmic of animals kept outdoors or indoors. Note the diurnal activity and high amplitude in the animals kept outdoors compared with the animals held indoors.

Their diurnal activity pattern is accompanied by anatomical and physiological adaptations. For example, they have higher epidermal melanin content and a remarkably cone-rich retina (41% of total photoreceptors in both central and peripheral retina) that is adapted to daylight vision (Djeridane, 1996; Saidi et al., 2011; Barak and Kronfeld-Schor, 2013). However, when transferred from the field or from outdoors colonies to the laboratory, fat sand rats lose their diurnal activity rhythm and become either nocturnal or arrhythmic (Figure 2).

The fat sand rats physiology, including reproduction, osmoregulation and energetics, was studied extensively in the laboratory (Degen et al., 1991; Fichet-Calvet et al., 1999; Haim et al., 2006). Moreover, fat sand rats were used in biomedical research for the study of the Metabolic Syndrome (Zimmet et al., 1999; Walder et al., 2002), including obesity (Collier et al., 1997a; Walder et al., 1997; Walder et al., 2000), type 2 diabetes mellitus (T2DM) and its complications (Kuwabara and Okisakaa, 1976; Collier et al., 1997b; Saïdi et al., 2011; Kaiser et al., 2012; Sahraoui et al., 2016), and fatty liver disease (Maislos et al., 2005; Zigmond et al., 2014), as well as seasonal affective disorder (Einat et al., 2006; Ashkenazy et al., 2009a; Ashkenazy et al., 2009b; Krivisky et al., 2011; Kronfeld-Schor and Einat, 2012). Therefore, many aspects of its physiology and behavior are well studied.

The fat sand rat has numerous attributes that make it an excellent model to study prevalent human health disorders with a circadian component such as Type 2 diabetes mellitus, obesity, cardiometabolic and mental health conditions such as depression and anxiety (see Table 1).

**TABLE 1:** Common disease manifestations of T2DM in fat sand rats and in humans.

Disorder	Fat Sand Rats with T2DM	Humans with T2DM
Elevated blood sugar	Yes	Yes
Obesity	Yes	Yes
Cataracts	Yes	Yes
Fatty liver	Yes	Yes
Cardiac hypertrophy and fibrosis	Yes	Yes
Depression	Yes	Yes
Circadian dysrhythmia	Yes	Yes

### Circadian desynchrony and type 2 diabetes in the fat sand rat

Studies from the 1960s demonstrated that when fat sand rats are kept under laboratory conditions and fed standard rodent diet (SRD), they develop T2DM within 4 weeks (Schmidt-Nielsen et al., 1964; Haines et al., 1965; Suckow et al., 2012). The diabetes ranges from mild hyperglycemia with hyperinsulinemia to hypoinsulinemia with ketoacidosis, a terminal stage with short survival (Suckow et al., 2012). As a result, the fat sand rat quickly became a frequently used, unique animal model for the study of diet-induced T2DM. Unlike most other animal models used for T2DM research, fat sand rats display a range of phenotypic characteristics when given free access to SRD. As might be expected for non-inbred progeny, adult fat sand rats have a wide range of body weight and body fat content that forms a continuous distribution (Walder et al., 2000). The development of diabetes in fat sand rats is gradual (Kalderon et al., 1986) and follows several defined stages. Some animals remain normoglycemic and normoinsulinemic (stage A), others are hyperinsulinemic and normoglycemic (stage B), and some develop overt insulin-resistant diabetes with hyperinsulinemia (stage C). As well, a small percentage develop, as a consequence of the long-lasting hyperglycemia, severe decompensated diabetes, loss of beta cells and hypoinsulinemia (stage D) (Kalderon et al., 1986). Fat sand rats are considered hyperglycemic when non-fasted blood glucose levels exceed 200 mg/dl, and considered hyperinsulinemic when non-fasted plasma insulin levels exceed 150µU/ml (Kalderon et al., 1986). The speed of progression from stage A to the other stages depends on the amount of digestible energy in the diet. When fed SRD, fat sand rats progress from stage A to stage C within 7–28 days (Kalderon et al., 1986).

Surprisingly, in a literature survey we undertook (Weir, 1974; Nakama, 1977; Shafrir and Adler, 1983; Ardiles et al., 2013; Subramaniam et al., 2018), we found that when transferred from the field to the laboratory and fed SRD, many diurnal rodents examined to date also develop T2DM, regardless of their natural diet (which is not necessarily low in caloric density). This effect was demonstrated in golden spiny mice (Shafrir and Adler, 1983), Nile grass rats (Subramaniam et al., 2018), degus (Ardiles et al., 2013), tuco-tuco (Weir, 1974) and Mongolian gerbil (Nakama, 1977). This change occurs in parallel to a switch of activity rhythm from diurnal to nocturnal, or the loss of a defined activity rhythm whatsoever (Umezu et al., 1989; Blanchong et al., 1999; Cohen and Kronfeld-Schor, 2006; Hagenauer and Lee, 2008; Tomotani et al., 2012; Barak and Kronfeld-Schor, 2013; Touati et al., 2018). This finding has led us to hypothesize that it is actually the disruption of circadian rhythms that accompanies the transfer to the laboratory that causes the development of T2DM in diurnal species, and that it is not necessarily the change in diet itself alone.

Disturbed circadian rhythms are becoming increasingly evident as linked to the etiology and pathophysiology of T2DM and cardiometabolic disorders (Masri and Sassone-Corsi, 2018; Zimmet et al., 2019). Epidemiological studies found that shift work increases the risk of T2DM compared with non-shift-workers (Gan et al., 2015), with a 5% increase in risk for every 5 years of the shift work (Pan et al., 2011). Rotating shifts, which disturb circadian rhythms even further, increase this risk even more as compared to night shifts (Pan et al., 2011). It was also suggested that disrupted/deficient sleep cycles exert an increased risk for T2DM, and sleep loss negatively impacts the treatment and management of T2DM in those individuals already diagnosed (Rakshit et al., 2014). The prevalence of T2DM in individuals with more than 1 hour of social jet lag (quantified as discrepancies in sleep/wake timing between work and free days (Wittmann et al., 2006)) is 1.75 times higher than that of people with less than 1 hour of social jet lag (Koopman et al., 2017). Moreover, late chronotypes, who are late risers with a more nocturnal activity pattern (Lack et al., 2009; Bauducco et al., 2020), have an odds ratio of about two to 2.5 fold for T2DM as compared with earlier chronotypes (Finn et al., 2013; Merikanto et al., 2013).

One of the most pronounced rhythmic aspects of physiology involves the daily variation of glucose tolerance and insulin sensitivity across the diel cycle (Polonsky et al., 1988). Disruption of this daily oscillation of glucose metabolism is a hallmark of T2DM (Polonsky et al., 1988). In individuals with T2DM, there is a daily rhythm in insulin sensitivity, with decreasing insulin sensitivity and increasing hepatic glucose production across the night (Boden et al., 1996), which leads to a fasting/morning hyperglycemia (Radziuk and Pye, 2006). Pinealectomy or bilateral sympathetic denervation of the pineal gland has been shown to significantly reduce insulin levels and increase blood glucose levels (Barragán et al., 1984; Munoz Barragán et al., 1986; LIMA et al., 2001; Peschke et al., 2015). SCN lesions in rats have been shown to abolish the daily rhythms in plasma concentrations of glucose and insulin (Yamamoto et al., 1987; la Fleur et al., 1999), and genetic disruption of clock genes in mice, such as in *Clock* (Rudic et al., 2004; Turek et al., 2005; Marcheva et al., 2010) and *Bmal1* (Rudic et al., 2004; Marcheva et al., 2010) mutant mice, cause hyperglycemia, hypoinsulinemia, and altered adipogenesis and hepatic carbohydrate metabolism. Interestingly, other studies demonstrate that SCN-lesioned rats and animals with a genetic disruption of the circadian clock show enhanced insulin sensitivity and glucose tolerance, supporting the role for the SCN in insulin-independent glucose uptake (la Fleur et al., 2001; Lamia et al., 2008; Jouffe et al., 2022) and complex relationships between chronodisruption and glucose metabolism. Moreover, the daily rhythm in the secretion of insulin from pancreatic islets is clock-driven (Boden et al., 1996; Peschke and Peschke, 1998; Tan and Scott, 2014; Peschke et al., 2015), and alterations in pancreatic clock gene expression such as CLOCK, BMAL1, CRY, PER1 and PER2, impair insulin secretion through predisposition to beta-cell failure (Marcheva et al., 2010; Rakshit et al., 2014; Vieira et al., 2014).

Additional support for the role of the circadian system in glucose homeostasis comes from our studies with the fat sand rats. Testing the effects of photoperiod (3 weeks of short photoperiod, SP, 5:19 L:D cycle–or neutral photoperiod: 12:12 L:D cycle, NP, as control) in combination with SRD or low energy diet (LED) *ad libitum* we found that male fat sand rats kept under SP were arrhythmic while fat sand rats kept under NP were nocturnally active. Animals kept under SP with SRD had significantly lower glucose tolerance and higher plasma insulin levels then all other groups. *Per2* mRNA showed a significant daily rhythm in NP acclimated animals, but not in SP acclimated animals, in both the SCN and the kidney (Bilu et al., 2019a). Interestingly, arrhythmic animals showed higher blood glucose levels and cholesterol levels compared with rhythmic animals (Bilu et al., 2022a). Comparing male and female fat sand rats maintained in SP or NP and fed SRD we found that the response differs between sexes: males kept under SP showed lower glucose tolerance and increased plasma insulin and cholesterol levels compared with females. Out of the four experimental groups, only NP acclimated females had a daily rhythm in blood glucose levels (Bilu et al., 2022a).

Our findings, supporting the link between modern lifestyle and circadian disruption and risk and etiology of T2DM, may have key implications for non-pharmacological prevention and therapeutic strategies to manage the contemporary and escalating T2DM epidemic occurring in humans (see below).

### Type 2 diabetes complications in the fat sand rat

Some of the well-described consequences of T2DM include the development of cardiovascular diseases, adipocyte dysfunction and cataracts (Hajer et al., 2008; Gregg et al., 2016; Glovaci et al., 2019). These consequences were also evident in the fat sand rats:

Cardiovascular diseases. Cardiovascular diseases are the leading cause of mortality in the United States, and ischemic heart disease accounts for 16% of all deaths around the world (Meléndez-Fernández et al., 2021). People with T2DM have a 2-to 4-fold increased risk of CVD events relative to those without diabetes (Fox et al., 2004). Due to increased atherosclerosis, hypertension is approximately twice as frequent in patients with diabetes compared with patients without the disease (Sowers et al., 2001). It may be present at the time of diagnosis or even before diabetes is diagnosed (Sowers et al., 2001). Diabetes is also associated with cardiac fibrosis, which reduces myocardial compliance, contributes to the pathogenesis of heart failure, and triggers arrhythmic events.

In our studies, we found that fat sand rats kept under SP regimen, regardless of diet, had significantly higher blood pressure than those under NP (Bilu et al., 2019a). It also resulted in an increased heart/body weight ratio in females, but not in males (Bilu et al., 2022a). Comparing rhythmic and a-rhythmic individuals, we found that regardless of sex, arrhythmic animals showed a higher heart/body weight ratio compared with rhythmic animals (Bilu et al., 2019b). Furthermore, regardless of treatment, diabetic animals exhibited a higher heart/total body weight ratio than the non-diabetic ones with a significant correlation between the heart/total body weight ratio and blood glucose levels in the oral glucose tolerance test (OGTT) (Bilu et al., 2019b). These phenotypic changes were also manifested at the molecular level: we found that animals kept under SP and fed SRD had reduced expression of the collagen gene Col1a1 in their cardiomyocytes, compared with animals kept under NP and fed LED. Furthermore, cardiomyocytes of SP acclimated animals expressed increased hypertrophy markers compared to animals kept under NP. Interestingly, inflammatory genes showed a daily rhythm only in animals kept under NP, suggesting a possible link between the circadian system and the pathology (Nankivell et al., 2021). We also found that SP animals, regardless of diet, showed a significant increase in perivascular fibrosis with higher levels of collagen deposition around myocardial vessels compared with NP acclimated animals fed LED. Histological analyses revealed that fat sand rats exposed to SP regimen with SRD had significantly smaller cardiomyocytes when compared to all other groups. In addition, NP acclimated animals fed SRD had significantly lower expression of the anti-apoptotic gene *Bcl2* in their cardiomyocytes (Nankivell et al., 2021). Together, our findings in fat sand rats suggest a relationship between the development of T2DM and cardiac pathology, similar to that seen in T2DM in human subjects (Crnko et al., 2019; Shi et al., 2021), and points to the possible involvement of the circadian system in the pathology.

*Cataracts.* Type 2 diabetes can affect all ocular structures, with cataracts being the most common ocular complication (Kiziltoprak et al., 2019). Cataracts in patients with diabetes are a major cause of blindness worldwide (Kiziltoprak et al., 2019). Diabetes is associated with an approximately two-fold increased detection rate of cataract (Becker et al., 2018). Cataracts are caused by generation of polyols from glucose by the enzyme aldose reductase. This results in increased osmotic stress in the lens fibers leading to their swelling and rupture (Pollreisz and Schmidt-Erfurth, 2010). Similar pathologies were also found in diabetic fat sand rats: 37% of SP acclimated animals developed cataracts, and none in fat sand rats kept under NP regimen (Bilu et al., 2019a). These findings suggest that the fat sand rat can also be a very useful animal model for the study of diabetic cataracts.

*Adipocyte dysfunction.* The adipose tissue has an important role in controlling whole-body glucose homeostasis in both normal and disease states (Guilherme et al., 2008). Dysfunctional adipose tissue phenotype underpins T2DM development (Tan et al., 2019) and is characterized by insulin-resistant adipocytes, adipocyte hypertrophy and a pro-inflammatory environment. Visceral adiposity and its white single-lipid-like adipocytes is known to accelerate the development of T2DM (Tan et al., 2019).

In order to determine if diet and/or photoperiod length influenced adipocyte dysfunction, we assessed the average expression and daily rhythm of key mediators involved in adipogenesis in the fat sand rats. We found that subcutaneous expression of adipocyte differentiation/function markers and transcription factors that regulate genes in glucose and lipid metabolism were reduced in animals kept under SP, fed SRD, or both. Furthermore, the average expression of visceral cytokines and adipose tissue browning markers showed significant circadian rhythms only in animals kept under NP conditions. We further found that animals fed SRD showed a significant increase in the area of visceral and subcutaneous adipocytes compared with LED-fed animals. Visceral adipocytes were also larger in animals acclimated to SP and fed SRD compared with other groups. In contrast, exposure to SP alone resulted in larger subcutaneous adipocytes compared to NP-acclimated animals fed LED. For both visceral and subcutaneous depots, animals acclimated to SP, fed SRD, or both, exhibited a higher proportion of larger adipocytes (Tan et al., 2019). Our research findings provide mechanistic insight on the possible influence of disrupted circadian rhythms on adipose tissue phenotype.

### The circadian clock, depression and T2DM

Significant data in humans and in animal models clearly demonstrate a bidirectional relationship between circadian rhythms and depression (Kronfeld-Schor and Einat, 2012; Walker et al., 2020a). People with genetic circadian disorders such as delayed sleep phase syndrome (Abe et al., 2011), familial advanced phase sleep syndrome (Jones et al., 1999; Xu et al., 2005) and acquired disorders such as shift-work sleep disorder (Kalmbach et al., 2015; Walker et al., 2020a) and chronic jet leg (i.e. people who regularly cross multiple time zones, such as international flight crews) have an increased risk for depression (Walker et al., 2020a; Walker et al., 2021). Disturbances of sleep-wake cycles are major core symptoms of depressive disorders; nearly constant symptoms of sleep disorders, as well as altered patterns of sleep architecture, have largely been described in depressed patients (Mendlewicz, 2009; Courtet and Olié, 2012). Furthermore, depressed patients show diurnal variations of symptoms, with the majority showing an increase in the intensity of symptoms in the early morning (Tolle and Goetze, 1987). Moreover, treatments targeting circadian rhythms such as sleep deprivation, bright light therapy (BLT), and behavioral therapies have been implicated as effective, indicating that depression is associated with alterations in circadian rhythms (Wirz-Justice et al., 2005), and antidepressant drugs normalize depression-related changes in sleep (Kronfeld-Schor and Einat, 2012). In mice, circadian disruption by acute or chronic exposure to light at night was shown to increase depressive- and anxiety-like behaviors in the open field test, elevated plus maze (EPM), and forced swim test (FST) compared to mice housed in dark nights. Nighttime light exposure was associated with reduced brain derived neurotrophic factor (BDNF) mRNA (Walker et al., 2020b). Reduced BDNF has also been linked to depression. In diurnal Nile grass rats chronic dim daylight intensity resulted in higher depression- and anxiety-like behaviors in the FST and in the sweet solution preference (SSP) test, as well as impaired spatial learning and memory in the Morris water maze (Yan et al., 2019).

Ample evidence in humans suggest a comorbidity between diabetes and depression, which is twice as frequent in people with T2DM compared with non-diabetic individuals (Sartorius, 2018; Prigge et al., 2022). This comorbidity was demonstrated in many studies and summarized in a large number of meta-analysis publications (Roy and Lloyd, 2012; Pashaki et al., 2019; Simayi and Mohemaiti, 2019). Yet, despite the overwhelming evidence for this comorbidity, its underlying mechanisms are not clear. In our studies, we found that male fat sand rats kept under SP showed increased anxiety and depression-like behavior in a number of relevant tests (Einat et al., 2006; Ashkenazy et al., 2009a; Bilu et al., 2019a). Diet, however, had no effect on the behavioral tests (Bilu et al., 2022a). Comparing diabetic *vs.* non-diabetic fat sand rats, we found that diabetic animals exhibited increased depressive and anxiety-like behaviors in the EPM and FST than non-diabetic ones. Moreover, depressive- and anxiety-like behaviors significantly correlated with blood glucose levels (Bilu et al., 2019b). Comparing males to females, we found that although in both sexes SP acclimation resulted in elevated depressive-like behavior in the FST compared to NP acclimation, this effect was more pronounced in males than females. Furthermore, in both sexes, arrhythmic animals showed increased depressive-like behavior than rhythmic animals (Bilu et al., 2022a). Similar results were obtained when instead of exposing the animals to short photoperiod, they received a daily intraperitoneal injection of either 100 µg melatonin (or vehicle solution as control), twice daily, 5 and 8.5 h after the onset of light in the room. These administration hours were chosen to mimic the effects of the SP cycle in the animals housed under NP conditions, as the half-life of exogenous melatonin is about 3.5 h (Hiebert et al., 2006)^,^ (Ashkenazy et al., 2009a).

All in all, we suggest that similar to the development of T2DM, the tendency of fat sand rats to develop depression- and anxiety-like behavior under laboratory conditions stems from disturbances in their circadian rhythms, which explains the comorbidity in the fat sand rats and may also be the underlying mechanism for the reported comorbidity in humans.

### BDNF

Significant data from humans and animal models indicate interactions between affective disorders and reduced plasticity of neuronal systems, suggesting a central role for BDNF and TrkB in the pathophysiology and treatment of these disorders (Bilu et al., 2022b). Low circulating BDNF levels were suggested as a biomarker for major depression, and increase in their levels is indicative of successful treatment of mood disorders (Karege et al., 2002; Giese et al., 2014; Allen et al., 2015; Polyakova et al., 2015; Bilu et al., 2022b). A perturbed or abnormal BDNF daily rhythm can influence neural circuits involved in affective disorders (Karege et al., 2002; Giese et al., 2014; Allen et al., 2015; Polyakova et al., 2015; Bilu et al., 2022b).

Human and animal studies suggest BDNF and TrkB, are also involved in the regulation of energy balance, glucose homeostasis, and the development of T2DM (Krabbe et al., 2007; Rosas-Vargas et al., 2011). Aberrant BDNF signaling in the brain triggers obesity and T2DM in mice (Marosi and Mattson, 2014). Patients with T2DM exhibit low plasma BDNF levels, which are inversely correlated with plasma glucose levels (Krabbe et al., 2007). It was shown that BDNF treatment reduces food intake, decrease non-fasting blood glucose levels and HbA1c and increases pancreatic insulin. Further, it is has been suggested that BDNF affects glucose metabolism not only in central metabolic pathways but also in peripheral glucagon secretion pathways (Eyileten et al., 2017).

Interestingly, BDNF is also involved in the function of the circadian system and its response to light (Liang et al., 2000; Allen et al., 2005).

In our studies with fat sand rats, we compared rhythms in plasma BDNF as well as BDNF and PER2 expression in the frontal cortex and SCN of males acclimated to SP and NP. Animals kept under NP exhibited a significant daily rhythm in plasma BDNF levels with higher levels during the night, and in BDNF expression levels in the frontal cortex and SCN. No significant BDNF rhythm was found in the plasma, frontal cortex or SCN of SP acclimated animals, which also had higher insulin levels, significantly higher glucose levels in the OGTT, and significantly higher anxiety- and depression-like behaviors compared with animals acclimated to NP (Bilu et al., 2022b).

Transcription rates of both Per2 and P75 ^NTR^, one of the receptors of BDNF, are controlled by the heteromeric Clock:Bmal1 transcription factor, which is part of the central clock mechanism, and their phases are synchronized (Baeza-Raja et al., 2013). P75^NTR^ participates in multiple intracellular signaling pathways to regulate a wide range of biological functions, including the regulation of glucose homeostasis and insulin sensitivity (Baeza-Raja et al., 2012). Insulin-stimulated glucose uptake is mediated by the glucose transporter 4 (GLUT4). In response to insulin signaling, GLUT4 is translocated from its intracellular compartment to the cell membrane. An increase in P75^NTR^ leads to a decrease in GLUT4 translocation to the cell membrane in response to insulin, resulting in lower glucose uptake (Baeza-Raja et al., 2012). A daily rhythm in P75^NTR^ is therefore expected to result in daily rhythm in insulin-stimulated glucose uptake. It is therefore possible that changes in the expression levels of BDNF and its receptor P75^NTR^ contributed to the development of glucose intolerance and high insulin levels in the SP acclimated animals.

Considering the involvement of BDNF in T2DM, affective disorders and the circadian system, our findings may suggest an underlying mechanism related to the comorbidity between circadian rhythms disturbances and these disorders.

### The circadian syndrome

The comorbid relationship between circadian rhythms disruption and the major components constituting the Metabolic Syndrome, has led us to propose that the Metabolic Syndrome can, at least in some of the cases, termed the Circadian Syndrome (Zimmet et al., 2019). The concept of the Circadian Syndrome (CircS) is built on the fact that a number of chronic disorders including obesity, hypertension, CMD, dyslipidemia, T2DM, depression, sleep disorder (eg sleep apnoea) and nonalcoholic fatty liver disease have a strong link with circadian rhythms (Maury et al., 2010; Shanmugam et al., 2013; Yohannes et al., 2013; He et al., 2016; Lemmer and Oster, 2018; Shetty et al., 2018; Bishehsari et al., 2020; Hernández-García et al., 2020). Despite the concept of CircS, no international definition of CircS has been proposed so far (Zimmet et al., 2019). It has been suggested that the CircS should be considered as a novel CVD risk cluster (Zimmet et al., 2019). In a recent study (Shi et al., 2021), we examined data regarding BMI, waist circumference, blood pressure, fasting blood glucose levels, lipid profile, sleep duration and depressive symptoms of 9,360 Chinese adults aged ≥40 years from the 2011 China Health and Retirement Longitudinal Study (CHARLS) (Zhao et al., 2014), and found that the CircS is a strong and better predictor for CVD than the Metabolic Syndrome in Chinese adults (Shi et al., 2021) and we are exploring this finding further in data bases from other communities.

### Chronotherapeutics

Chronotherapeutics is the application of circadian principles in treating circadian rhythm-related disorders (Wirz-Justice, 2009). It is currently most used in the field of mood and sleep disorders (Geoffroy and Palagini, 2021).

*Bright light treatment.* Light therapy is recommended as a first-line monotherapy for all depression subtypes (Geoffroy and Palagini, 2021). It is first of all a circadian intervention; It can shift phase, increase amplitude, and stabilize daily rhythms (Wirz-Justice, 2009). Light synchronizes the SCN by means of a neuronal tract from a group of specialized ganglion cells in the retina containing the photopigment melanopsin, which is primarily sensitive to blue-wavelength light (Hankins et al., 2008). This photic input is separate from, but interacts with, the rods and cones of the retina, and informs the SCN whether it is dawn or dusk, light or dark (Hattar et al., 2003). Light in the morning advances the timing of the SCN clock, whereas light in the evening delays it. Thus, subtle shifts induced by light exposure at the twilight transitions resets the internal clock to a strict 24-h rhythm (Wirz-Justice, 2009; Zhu and Zee, 2012). For example, in diurnal four-striped mice, higher daytime light intensity increased the amplitude of the SCN’s daily peak in spontaneous firing rates and neuronal depolarization and enhanced the amplitude of its daily rhythm in spontaneous activity. This was associated with improvements in daily rhythms of general activity, wheel running and body temperature (Beatriz et al., 2021).

Evidence suggest that light treatment has antidepressant-like effects on all depression subtypes (Geoffroy and Palagini, 2021). The most studied example of the alleviating effect of light treatment on depressive symptoms is that of seasonal affective disorder (SAD) (Nieuwenhuis et al., 2009). Treatment generally involves daily administration of bright artificial light (BLT) indoors, most often at home within outpatient protocols (Terman et al., 1989). Most studies have used full-spectrum light of approximately 2,500–3,000 lux illuminance (an intensity obtained outdoors within a few minutes of sunrise) for 30–120 min daily in the morning, at a distance of about 1 m from the eyes (Terman et al., 1989). In animal models such as Mongolian gerbils, BLT was shown to improve depression- and anxiety-like behaviors in the EPM and FST in animals exposed to chronic mild stress, a validated method to induce depression-like behavior (Willner, 1997). In humans also, reviews and meta-analyses have confirmed the efficacy of light treatment for seasonal affective disorder, with a clinical response rate of approximately 65% (Terman et al., 1989; Tam et al., 1995; Lee et al., 1997; Levitt and Lam, 1999; Thompson et al., 2001; Magnusson and Boivin, 2003).

Based on the hypothesis that circadian rhythms disruption accelerates the development of T2DM, and that BLT acts, at least partially, through the entrainment of the circadian system (Satlin et al., 1992), a few studies tested the effect of BLT on insulin sensitivity in patients with T2DM. These concluded that BLT may be a promising treatment for a subgroup of highly insulin resistant individuals with T2DM (Brouwer et al., 2019). Moreover, two separate case reports found that BLT increased insulin sensitivity in T2DM patients (Allen et al., 1992; Nieuwenhuis et al., 2009).

Our studies with fat sand rats further support the efficacy of BLT for the treatment of circadian-rhythm related diseases. Fat sand rats treated with BLT show reduced anxiety-like behavior in the EPM as well as reduced depression-like behavior in the FST, compared with non-treated animals (Ashkenazy et al., 2009b). Fat sand rats kept under NP or SP as well as animals kept under SP with BLT were arrhythmic or nocturnal whereas animals kept under NP with BLT were mostly diurnal. The SP group with no BLT had a significantly higher number of arrhythmic animals compared with all other groups. Interestingly, short photoperiods resulted in higher fasting glucose levels, whereas BLT resulted in reduction in glucose levels. Similar effects were demonstrated in the glucose tolerance test, with higher blood glucose levels in animals maintained at SP and lower levels in animals that were exposed to BLT.

Furthermore, only fat sand rats receiving BLT showed a daily rhythm of blood glucose levels. Bright light treatment also reduced heart weight and heart/body weight ratio, regardless of photoperiod. Moreover, only BLT-treated animals showed daily rhythms in *Per2* gene expression in the SCN. *Per2* gene expression in the PFC, liver and kidney exhibited a daily rhythm only in animals acclimated to NP with BLT treatment, further supporting our hypothesis of the circadian rhythms disturbance involvement in the development of the comorbidity of depressive-like behavior and T2DM in the fat sand rats (Bilu et al., 2020).

We also found that as reported in humans, morning BLT has a stronger effect compared to evening BLT: it decreased anxiety and depression-like behavior in the EPM and FST whereas evening bright light was effective only in the FST. Comparing the effect of morning BLT at different wavelength [wide-spectrum light (3,000 lux, wavelength 420–780 nm, 5487 K), blue (1,300 lux, wavelength 420–530 nm) and red light (1,300 lux, wavelength range 600–780 nm)] we found that both full spectrum and blue light exposures decreased depression-like behavior in the FST and FST compared with red light exposure and no exposure groups (Bilu et al., 2019c), supporting the involvement of the melanopsin photoreceptor and the circadian system in the effect.

Physical exercise. Physical exercise synchronizes the circadian system and has ameliorating effects on the depression- and anxiety-like phenotype induced by circadian disruption in mice, as well as on depression symptoms in humans (Cooney et al., 2013). Voluntary wheel running in mice exerts faster recovery of internal synchrony following light/dark shift, and increases amplitude of SCN firing rates (Solberg et al., 1999; Kas and Edgar, 2001; Castillo et al., 2011). The effects of exercise on the circadian system may be mediated through skeletal muscles (Hower et al., 2018). Skeletal muscle and bone control nutritional homeostasis, such as maintaining glucose and calcium levels. Feeding and exercise stimulate skeletal muscle tissues and change their functions, including the maintenance of tissue mass and metabolism (Aoyama and Shibata, 2017). Moreover, physical exercise elevates the arterial CO_2_ tension, and changes in carbon dioxide levels can alter the expression of several core circadian clock genes and phase shift circadian rhythmicity in cultured cells (Adamovich et al., 2019). Through these interactions with skeletal muscles and by inducing an elevation in arterial CO_2_ tension, exercise may regulate circadian factors that influence mental, metabolic, and cardiovascular health. For instance, deregulated circadian rhythms in skeletal muscles are associated with reduced glucose tolerance, as well as increased rates of diabetes and CVD (Harfmann et al., 2015; Schroder et al., 2015).

Two of our studies examined the effect of voluntary exercise on daily rhythms in fat sand rats. In the first study (Tal-Krivisky et al., 2015), we found that in contrast to controls, all animals with access to running wheels showed significant activity rhythms both under NP or SP. The amplitude of the general activity in the SP groups was tripled in the group which had access to the running wheel. The availability of running wheels significantly decreased anxiety-like behavior in the EPM in animals maintained under SP but did not affect animals kept under NP. The same effect was observed for depression-like behavior in the FST. In a second experiment (Bilu et al., 2022c), we found that fat sand rats kept without access to running wheels were all arrhythmic, whereas animals kept with running heels were either diurnal (5/10), nocturnal (2/10), or arrhythmic (3/10). Fat sand rats without running wheels showed significantly higher blood glucose levels than the ones with wheels, both at baseline and 120 min after oral glucose administration in the OGTT. Only fat sand rats with access to running wheels showed a daily rhythm in blood glucose levels. Accordingly, plasma insulin was significantly higher in animals kept with no running wheels compared with animals kept with running wheels. Furthermore, the liver weight, heart weight and heart/body weight ratio were larger, and the left ventricular wall was thicker in fat sand rats with access to running wheels compared to fat sand rats with no running wheels. Animals kept with no access to running wheels showed increased anxiety- and depression-like behaviors in the EPM and FST, and decreased memory in the novel object recognition test, compared to animals kept with running wheels (Bilu et al., 2022c).

## Conclusion

In summary, we have shown that fat sand rats (*Psammomys obesus*) living under conditions mimicking modern lifestyle, have dampened behavioral and biological rhythms and develop circadian desynchrony that leads to depression- and anxiety-like behaviors, T2DM, obesity and CVD. The development of these disorders is accelerated under SP conditions (Madsen Ostercamp and Osterkamp, 1982; Bilu et al., 2019a; Bilu et al., 2019c; Tan et al., 2019; Nankivell et al., 2021; Bilu et al., 2022b) (see Table 2). We suggest that when environmental rhythmicity declines as a result of modern time living conditions in humans or laboratory conditions in diurnal sand rats, they do not entrain well and develop circadian rhythms related disorders. This makes the fat sand rats an excellent model of reversible circadian desynchrony for the studies of metabolic and other circadian rhythm related disorders.

**TABLE 2:** Comparison of physiological and behavioral measures of male and female fat sand rats under SP or NP.

Measure	Males			Females	
	NP	SP	Outdoors	NP	SP
Activity	Arrhythmic (Bilu et al., 2019a; Bilu et al., 2022a)	Arrhythmic (Bilu et al., 2019a; Bilu et al., 2022a)	Diurnal (Bilu et al., 2019a)	Arrhythmic (Bilu et al., 2022a)	Arrhythmic (Bilu et al., 2022a)
T2DM	No (Bilu et al., 2019a; Bilu et al., 2022a)	Yes (Bilu et al., 2019a; Bilu et al., 2022a)	No (Bilu et al., 2019a)	No (Bilu et al., 2022a)	Yes (Bilu et al., 2022a)
24-h glucose rhythm	Arrhythmic (Bilu et al., 2019a; Bilu et al., 2022a)	Arrhythmic (Bilu et al., 2019a; Bilu et al., 2022a)	Rhythmic (Bilu et al., 2019a)	Arrhythmic (Bilu et al., 2022a)	Rhythmic (Bilu et al., 2022a)
Insulin levels (ng/ml)	Normal (Bilu et al., 2019a; Bilu et al., 2022a)	High (Bilu et al., 2019a; Bilu et al., 2022a)	-	Normal (Bilu et al., 2022a)	High (Bilu et al., 2022a)
Cholesterol levels (mg/dl)	Normal (Bilu et al., 2019a; Bilu et al., 2022a)	High (Bilu et al., 2019a; Bilu et al., 2022a)	-	Normal (Bilu et al., 2022a)	Normal (Bilu et al., 2022a)
Body weight (g)	Lower than 240^49,125^	Higher than 260^49,125^	Lower than 240^49^	Lower than 240,^125^	Lower than 240,^125^
Blood pressure (mmHg)	Lower than 100^49^	Higher than 110^49^	-	-	-
Heart weight (g)	0.6^125^	0.6^125^	-	0.4^125^	0.5^125^
Heart/body weight ratio	0.0023^125^	0.0023^125^	-	0.0018^125^	0.0023^125^
Cataracts	No (Bilu et al., 2019a)	37% of animals (Bilu et al., 2019a)	No [unpublished]	No [unpublished]	No [unpublished]
24-h Per2 mRNA rhythm in SCN and kidney	Rhythmic (Bilu et al., 2019a)	Arrhythmic (Bilu et al., 2019a)	-	-	-
Depressive-like behavior	Low (Ashkenazy et al., 2009a; Bilu et al., 2019a; Bilu et al., 2022a)	High (Ashkenazy et al., 2009a; Bilu et al., 2019a; Bilu et al., 2022a)	-	Low (Bilu et al., 2022a)	High (Bilu et al., 2022a)
Anxiety-like behavior	Low (Ashkenazy et al., 2009a; Bilu et al., 2019a; Bilu et al., 2022a)	High (Ashkenazy et al., 2009a; Bilu et al., 2019a; Bilu et al., 2022a)	-	Low (Bilu et al., 2022a)	High (Bilu et al., 2022a)

The comorbidity between these pathologies and circadian rhythm disruption has led us to suggest that the ubiquitous Metabolic Syndrome should at least in some of the cases be renamed the “Circadian Syndrome”. This gives greater emphasis to the role of circadian desynchrony in the etiology of T2DM and CVD (Zimmet et al., 2019). We further demonstrate that environmental manipulations that increase rhythmicity, improve or prevent these pathologies (Ashkenazy et al., 2009b; Krivisky et al., 2012; Bilu et al., 2019c; Bilu et al., 2020; Bilu et al., 2022c). We suggest that using diurnal animal models to study circadian rhythm-related diseases such as depression, T2DM and CVD will produce new insights which will eventually lead to the development of more effective prevention and treatment strategies for people with cardiometabolic and mental health disorders and other conditions related to circadian disruption. Currently, these highly prevalent circadian rhythm-related disorders constitute a serious yet still poorly recognized public health challenge that imposes an exceptionally large health and economic burden globally. Together, they can threaten the future human health and wellbeing.
